# Daily rhythm of cerebral blood flow velocity

**DOI:** 10.1186/1740-3391-3-3

**Published:** 2005-03-10

**Authors:** Deirdre A Conroy, Arthur J Spielman, Rebecca Q Scott

**Affiliations:** 1 Department of Psychology, The Graduate School and University Center of the City University of New York, New York, USA; 2 Department of Neurology and Neuroscience, New York Presbyterian Hospital, New York, USA; 3 Department of Health Psychology, Albert Einstein Medical College at Yeshiva University, Bronx, USA

## Abstract

**Background:**

CBFV (cerebral blood flow velocity) is lower in the morning than in the afternoon and evening. Two hypotheses have been proposed to explain the time of day changes in CBFV: 1) CBFV changes are due to sleep-associated processes or 2) time of day changes in CBFV are due to an endogenous circadian rhythm independent of sleep. The aim of this study was to examine CBFV over 30 hours of sustained wakefulness to determine whether CBFV exhibits fluctuations associated with time of day.

**Methods:**

Eleven subjects underwent a modified constant routine protocol. CBFV from the middle cerebral artery was monitored by chronic recording of Transcranial Doppler (TCD) ultrasonography. Other variables included core body temperature (CBT), end-tidal carbon dioxide (EtCO2), blood pressure, and heart rate. Salivary dim light melatonin onset (DLMO) served as a measure of endogenous circadian phase position.

**Results:**

A non-linear multiple regression, cosine fit analysis revealed that both the CBT and CBFV rhythm fit a 24 hour rhythm (R^2 ^= 0.62 and R^2 ^= 0.68, respectively). Circadian phase position of CBT occurred at 6:05 am while CBFV occurred at 12:02 pm, revealing a six hour, or 90 degree difference between these two rhythms (t = 4.9, df = 10, p < 0.01). Once aligned, the rhythm of CBFV closely tracked the rhythm of CBT as demonstrated by the substantial correlation between these two measures (r = 0.77, p < 0.01).

**Conclusion:**

In conclusion, time of day variations in CBFV have an approximately 24 hour rhythm under constant conditions, suggesting regulation by a circadian oscillator. The 90 degree-phase angle difference between the CBT and CBFV rhythms may help explain previous findings of lower CBFV values in the morning. The phase difference occurs at a time period during which cognitive performance decrements have been observed and when both cardiovascular and cerebrovascular events occur more frequently. The mechanisms underlying this phase angle difference require further exploration.

## Background

It has been well documented that cerebral blood flow velocity (CBFV) is lower in sleep [1-7] and in the morning shortly after awakening [8-10] than in the afternoon or evening. Generally accepted theories about the time of day changes in CBFV attribute the fall in CBFV to the physiological processes of the sleep period and the increase during the day to waking processes. The low CBFV in the morning is thought to be a consequence of the fall in the overall reduced metabolic level [8,10,11] and reduced cognitive processing [12]. Additionally, the reduced physical activity [13], reduced body temperature, and the recumbent sleeping position have also been proposed as contributors [14] to the decline in CBFV and analogous brain processes.

An alternative to these explanations that attribute changes in CBFV to sleep and wake dependent processes is that this pattern of fluctuation reflects an endogenous process with circadian rhythmicity. The decline of CBFV across the sleep period and rise after subjects are awakened in the morning resemble the endogenous circadian changes in core body temperature (CBT), a reliable index of endogenous circadian rhythmicity. Both patterns are low during sleep, start to rise in the morning, reach their peak in the late afternoon, and then drop during the sleep period.

The aim of this study was to examine CBFV over ~30 hours of sustained wakefulness to unmask and quantify contributions of the endogenous circadian system. By not permitting sleep, the evoked changes dependent on this change of state will not contribute to the observed CBFV changes. We hypothesized that time of day changes in CBFV are due to endogenous circadian regulation. Previous studies have been limited by several factors. First, the environmental conditions (light level) and the behavior of the subject (sleep, meals, and caffeine intake) were not controlled [15,13,1,16]. Second, CBFV measurements were obtained at only a few circadian points. For example, Ameriso et al. [15] and Qureshi et al. [16] assessed CBFV between 6–8 am, 1–3 pm, and 7–9 pm. Diamant et al [13] assessed CBFV during the first 15 minutes of every hour across a 24 hour period. Given these brief time periods, the findings are only a schematic of the 24 hour profile. Third, primary output markers of the endogenous circadian pacemaker (such as core body temperature and melatonin production) were not assessed.

We employed the "constant routine" protocol, which was designed specifically to unmask underlying circadian rhythms in constant conditions [17]. CBFV was collected by Transcranial Doppler (TCD) ultrasonography for the entire study period. Core body temperature and salivary dim-light melatonin onset (DLMO) were measured for determination of circadian phase. Continuous electroencephalography (EEG) was performed to ensure wakefulness across the study. Additionally, measurements of blood pressure, heart rate, and end tidal carbon dioxide (Et_CO2_), three of the main regulators of CBFV, were collected every half hour.

## Methods

### Subject selection

Twelve subjects (10 men and 2 women; ages 19–38, mean 28 years) agreed to participate. One subject discontinued her participation because of a headache 15 hours into the study. Subjects were in good health, as assessed by medical history, semi-structured clinical interview, and physical exam. Information regarding menstrual cycle was not obtained from female subjects. Subjects also underwent an independent standard cerebrovascular assessment and were determined to be normal. They reported no symptoms of sleep problems (such as insomnia, obstructive sleep apnea, narcolepsy, or restless legs syndrome).

Subjects that were selected to participate kept to a designated sleep-wake schedule (that was negotiated from the subject's typical pattern) and filled out a sleep diary for the two weeks prior to the time in the laboratory. According to sleep diary reports, bedtimes ranged from 10:30 pm to 1:00 am and waketimes ranged from 6:00 am to 10:00 am. Alcohol and caffeine intake was discontinued for the entire week before the study. During the data collection, subjects were not permitted either alcohol or caffeine. All subjects were non-smokers.

### Laboratory constant routine protocol

The study protocol was approved by the Institutional Review Boards of New York Presbyterian Hospital – Weill Medical College of Cornell University and The City College of New York. Subjects gave written and informed consent before participating. Subjects arrived at the sleep laboratory between 9:30 am and 10:00 am. They were oriented to the study procedures and to their bedroom. Electrodes were placed on the subject's head and face as they sat in a chair next to the bed. Data collection began at 11 am. Subjects remained in bed and awake in a semi recumbent position for 30 hours in an established "constant routine" (CR) protocol. Subjects remained in low (<25 lux) light levels which have been shown to have little or no entraining effect on the circadian pacemaker [18]. They were not allowed to get out of bed to urinate. Instead they urinated in private in a urinal or bedpan. Subjects remained awake from 11:00 a.m. on Day 1 until 5 p.m. on Day 2. Throughout the study, subjects were provided small meals (Ensure ^® ^liquid formula plus one-quarter nutritional food bar) every 2 hours. Subject's typical total food and liquid intake for a day and a quarter were divided into 15 relatively equal portions. Only one subject participated in the CR per 30-hour period.

This protocol represents a modified CR in two ways. First, subjects were allowed to watch television and were therefore were not in "time isolation." Television content was monitored so that subjects were not exposed to programs with highly emotional themes. Second, subjects needing to defecate were allowed to go to the bathroom, which was located a few steps away from the bedside. We chose this method as an alternative to using the bedpan to ensure subject's comfort and study compliance. Three subjects (subjects 05, 06, and 10) got out of bed once at 3:30, 21:30, and 15:30, respectively, to defecate. One subject, subject 12, got out of bed twice, at 22:30 and 6:35. Subject 10 used the bathroom only during the adaptation period. A paired-samples t-test was conducted to evaluate the impact of getting out of bed to defecate on subject's CBT and CBFV values. The CBT and CBFV values in the two hours before getting up were compared to the two hours after the subject got up. Subjects 5 showed a slight decrease in CBT from before (M = 98.12, SD = 0.14) to after the subject returned to the bed (M = 97.91, SD = 0.08), t(3) = -5.17, p = .014). Subject 6 showed a decline in CBFV from before (M = 56.14, SD = 2.3) to after the subject returned to the bed (M = 45.67, SD = 3.7), t(3) = 5.49, p = 0.012). There were no other significant differences detected between these two time periods for subject 5's CBFV, subject 6's CBT, or for both times subject 12 got out of the bed. By visual inspection, the overall shape of the curves in these subjects was not affected and therefore these subject's data were included in subsequent analyses.

### Transcranial Doppler ultrasound recordings

The current study utilized TCD ultrasonography to measure cerebral blood flow velocity. TCD is a non-invasive instrument (consisting of one or two 2-Mhz transducers fitted to a headband, MARC500, Spencer Technologies, Nicolet Biomedical Inc) that is used predominantly as a diagnostic tool to assess cerebral hemodynamics in normal and pathological conditions. TCD ultrasonography is predicated on a theory that involves the measurement of moving objects when combined with radar. When the instrument emits the sound wave, it is reflected by the blood cells that are moving in the vector of the sound wave [19].

CBFV was measured using either the right or left middle cerebral artery (MCA) using TCD sonography (TCD: DWL Multidop X-2, DWL Elektronische Systeme GmbH, D-78354 Sipplingen/Germany) through the temporal window. An observer who was present continuously during the recordings evaluated the quality of the signal. This enabled long-term recording of CBFV throughout the study. Fast Fourier Transformation (FFT) of the signal was used to analyze the velocity spectra. The mean velocity of the MCA was obtained from the integral of the maximal TCD frequency shifts over one beat divided by the corresponding beat interval and expressed in cm/sec. Analysis was conducted off line.

### Measurement of standard markers of the circadian pacemaker

#### Body temperature recordings

Core body temperature was recorded at 1-minute intervals with an indwelling rectal probe (MiniMitter, Co. Bend, OR). A wire lead connected the sensor out of the rectum to a data collection system worn on the belt. Temperature readings were collected and saved into the device and monitored at hourly intervals by the investigator. After the study, the recordings were visually inspected and artifacts resulting from removal or malfunction of the probe were excluded from further analysis.

#### Salivary melatonin

Salivary samples of 3 ml were collected every hour from 11:00 a.m. on Day 1 to 4:00 p.m. on Day 2. Ten of these samples were used only for the determination of the timing of the salivary dim light melatonin onset (DLMO). For nine subjects, salivary DLMO was assessed across a ten-hour time window that included the ten hours before the CBT minimum. Immediately after collection, each saliva sample was frozen and stored at -20°C. Saliva samples were assayed using Bühlmann Melatonin Radio Immunoassay (RIA) test kit for direct melatonin in human saliva (American Laboratory Products Co., Windham, NH). Analysis was conducted at New York State Institute for Basic Research. Salivary DLMO time was selected based on two criteria. The saliva sample needed to have melatonin concentration 3 pg/ml or above and later samples needed to show higher levels (Bühlmann laboratories). Second, the 3 pg/ml threshold needed to occur within 6–10 hours before core body temperature minimum [20].

#### Polygraphic recordings

Electroencephalography (EEG) was continually assessed across the 30 hours to ensure that subjects maintained wakefulness. The following montage was used according to the international 10–20 system: C3-A2, C4-A1, O1-A2, O2-A1, ROC-A1, LOC-A2, and submentalis electromyogram (EMG). One channel of electrocardiogram was continuously recorded by monitoring from two electrodes (one on each side of the body at the shoulder chest junction). The EEG software (Rembrant Sleep Collection Software Version 7.0) was used for data acquisition and display of the signals on a personal computer. Throughout the CR, the investigator (DAC) monitored the quality of the recordings. The recordings were scored by RQS and DAC.

#### Blood pressure, heart rate, and end-tidal CO2

An automated blood pressure cuff was placed on the bicep of the subject and inflated two times each hour in order to determine changes in blood pressure and heart rate over time. Blood pressure and heart rate in one subject (02) was recorded via a finger blood pressure monitor (Omron Marshall Products, Model F-88). Blood pressure and heart rate in subjects 03, 04, 05, 06, and 07 were recorded with Omron Healthcare, Inc, Vernon Hills, Illinois 60061 Model # HEM-705CP Rating: DC 6V 4W Serial No: 2301182L. Blood pressure and heart rate for subjects 08, 09 and 10 was recorded with a similar blood pressure monitor (CVS Pharmacy Inc, Woonsocket, RI 02895 Model # 1086CVS). Blood pressure and heart rate recordings were not measured in subjects 11 and 12. Et_CO2 _was continuously obtained. A nasal cannula for monitoring expired gases was placed under the nose. Relative changes in carbon dioxide content were measured by an Ohmeda 4700 Oxicap (BOC healthcare). Mean Et_CO2 _levels were analyzed off-line. Et_CO2 _recordings were not measured in subjects 11 and 12.

### Data Analyses

#### Data reduction and statistical procedures

CBT and CBFV values were first subjected to data rejection. All CBT values less than 96 degrees were determined to be artifact and were rejected. All CBFV values less than 20 cm/sec were determined to be artifact according to the clinical criteria set by the staff neurologist. Data reduction was accomplished by averaging into one minute, 30 minute or hourly bins. Correlations presented here were performed on mean values in 30 minute bins. To ensure that circadian measurements were made under basal conditions, the first five hours of the constant routine were excluded from all analyses to eliminate effects of study adaptation. The last hour was excluded to eliminate confounding effects such as expectation effects.

**Figure 1:**
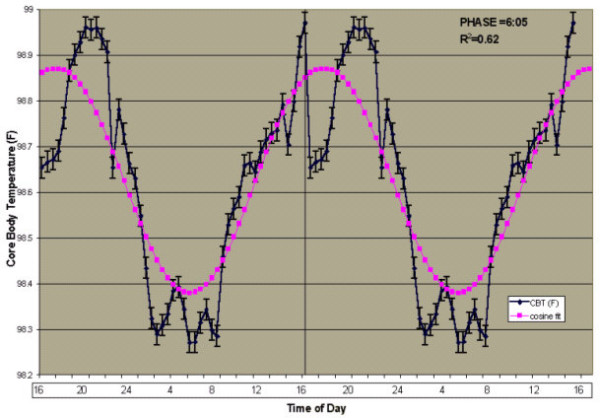
**24-hour Cosine Curve fit to Mean Core Body Temperature (°F). **Time course of CBT according to time of day. Shown is a double plot of the group (n = 11) mean levels (+/- SEM) of CBT (blue diamonds) fit with a 24-hour cosine curve (purple squares). Time of day is shown on the abscissa. The ordinate shows CBT values (degrees F). The vertical line indicates where the data was double plotted. Also displayed in the upper right corner is the non-linear cosine curve fit for mean CBT, R^2 ^= 0.62. The overall mean circadian phase position of the minimum was 6:05 am.

**Figure 2:**
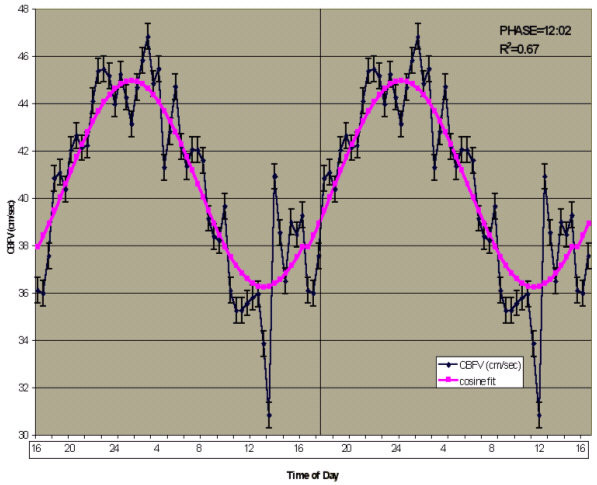
**24-hour Cosine Curve fit to Mean Cerebral Blood Flow Velocity (cm/sec). **Time course of CBFV according to time of day. Shown is a double plot of the group (n = 11) mean levels (+/- SEM) of CBFV (blue diamonds) fit with a 24-hour cosine curve (purple squares). Time of day is shown on the abscissa. The ordinate shows CBFV values (cm/sec). The vertical line indicates where the data was double plotted. Also displayed in the upper right corner is the non-linear cosine curve fit for mean CBFV, R^2 ^= 0.67. The overall mean circadian phase position of the minimum was 12:02 pm.

**Figure 3:**
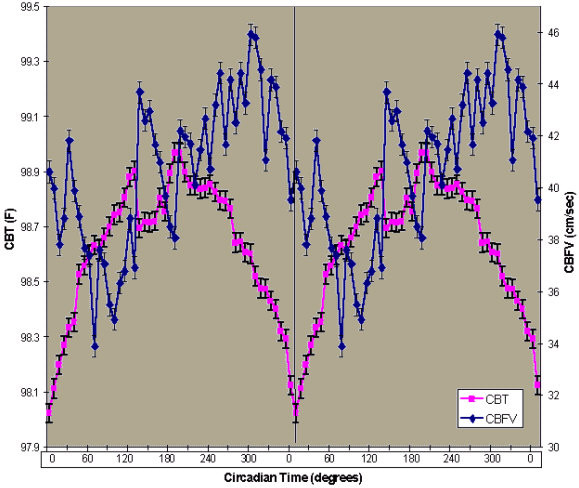
**Mean CBT and CBFV Aligned to CBT Nadir. **Time course of mean CBFV and mean CBT aligned to the nadir of CBT and then averaged. Shown is a double plot of the group (n = 11) mean levels (+/-SEM) of CBT (purple squares) and CBFV (blue circles) aligned to the phase of the circadian temperature cycle. Circadian time in degrees is shown on the abscissa. The ordinate on the left shows CBT values (degrees F) and CBFV (cm/sec) on the right. The vertical line indicates the CBT nadir.

**Figure 4:**
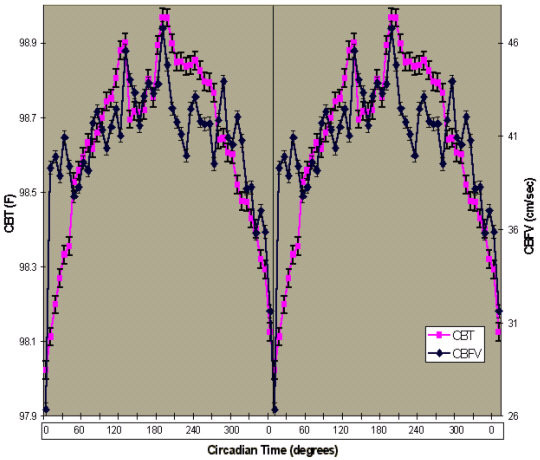
**Mean CBT and CBFV Aligned to Their Respective Nadir. **Time course of mean CBFV and mean CBT aligned to each of their respective nadirs and then averaged. Shown is a double plot of the group (n = 11) mean levels (+/-SEM) of CBT (purple squares) and CBFV (blue circles) aligned to the phase of the circadian temperature cycle. Circadian time in degrees is shown on the abscissa. The ordinate on the left shows CBT values (degrees F) and CBFV (cm/sec) on the right. The vertical line indicates both the CBT nadir and the CBFV nadir. The correlation coefficient between the aligned rhythms is 0.77 (p < 0.01).

The data are presented in this article in three ways. First, CBT and CBFV values were plotted according to time of day (Figures 1 and 2). Second, CBFV values were aligned according to the CBT nadir (Figure 3) and third, the CBFV nadir was aligned to the CBT nadir (Figure 4). To align CBFV to the CBT circadian nadir as shown in Figure 3, the CBT nadir of each individual subject was set to circadian time 0, or 0°. The CBFV value that corresponded to the CBT nadir was then also set to 0. Each half hour data point after the temperature nadir and corresponding CBFV values were then set to a circadian degree. There were a total of 48 data points across the 24 hour period. Therefore, each data point was equal to 7.5 degrees so that each data point would accumulate to 360°. Lastly, mean values were obtained for CBT and CBFV at each circadian degree.

To align the CBFV nadir to the CBT nadir, first, the lowest value of CBT and the lowest value of CBFV were identified and set to circadian time 0, or 0°. Each half hour data point after the CBT nadir and CBFV nadir were then set to a circadian degree. There were a total of 48 data points across the 24 hour period. Therefore, each data point was equal to 7.5 degrees so that each data point would accumulate to 360°. Lastly, mean values were obtained for CBT and CBFV at each circadian degree.

#### Estimation of circadian phase

A 24-hour non-linear multiple regression -cosine curve fit analysis was performed on the CBT and CBFV data (SAS Institute, Cary, NC). This technique constrains the circadian period of CBT and CBFV to be within 24 hours. This technique used the following equations: model cbt = &avg_cbt + r * cos((2 * 3.1415) * (hours-&max_cbt)/24; model cbfv = &avg_cbt + r * cos((2 * 3.1415) * (hours-&max_cbfv)/24, where & = constants that center the curve at the actual average for each series (vertical centering) and the predicted maximum at the actual maximum (horizontal centering); r = the amplitude of the cosine wave. An additional analysis was performed which also yielded the estimated clock time for the CBT nadir and CBFV nadir (Synergy software, Kaleidagraph Version 3.6). Third, the minimum of the circadian rhythm of CBT and salivary DLMO were also used as markers of the endogenous circadian phase. A paired t-test was used to determine the overall phase difference between CBT and CBFV.

## Results

Eleven subjects completed the protocol. The TCD probe was placed on either the right or left temple, whichever gave the better signal. Mean isonation depth of the TCD signal was 56.5 mm for the right MCA and 55.6 mm for the left MCA (range 53–60 mm). The constant routine ranged from 28 to 30 hours in duration. Polygraphic recordings confirmed sustained wakefulness across essentially the entire protocol in all but one subject. Subjects that had difficulty remaining awake were monitored closely and aroused when needed by engagement in conversation. Results from the polygraphic recordings are not presented here. We do not present the results of the polygraphic recordings because, for the purposes of this study, these recordings were used solely to monitor whether subjects were awake or asleep. The first five hours and the final hour of data from the constant routine were excluded from analysis.

### Core body temperature, cerebral blood flow velocity and the 24-hour day

A 24 hour non-linear multiple regression, cosine fit analysis revealed that the overall mean CBT rhythm (n = 11) fit a 24 hour cosine rhythm (R^2 ^= 0.62, p < 0.01), Figure 1. The mean CBT across all subjects was 98.6 °F (+/- 0.03 °F). Figure 2 shows that a 24-hour non-linear multiple regression, cosine analysis fit a 24 hour cosine rhythm (R^2 ^= 0.67, p < 0.01), Figure 2. The mean CBFV across subjects was 40.6 cm/sec (+/- 0.54 cm/sec). Salivary DLMO occurred 7.7 hours prior to the CBT nadir in nine subjects, which served only as a secondary measure of endogenous circadian phase position in those subjects. The mean salivary melatonin concentration across the ten hour window was 15.3 pg/ml (+/-3.05 pg/ml).

### CBFV rhythm is 90 degrees out of phase with the CBT rhythm

The overall mean circadian position of CBT occurred at 6:05 am and the mean position of CBFV occurred at 12:02 pm (Figure 3), yielding a 6 hour or 90 degree statistically significant difference (t = 4.9, DF = 10, p < 0.01). In individual subject data, the differences ranged from 0 to 8.5 hours. In eight subjects, the CBFV phase occurred later than the respective CBT phase, with mean difference of 5.2 hours. In two subjects, the CBFV nadir occurred earlier than the respective CBT nadir, with a mean difference of 6 hours. In one subject, there was no difference between the phase of CBT and CBFV. However, this subject's CBT rhythm was highly unusual, with the nadir occurring at 11:35 am on Day 2. Nevertheless, we felt the most appropriate way to present the data was to include this subject in the overall analysis. When the phase of CBFV was shifted so that the lowest value was aligned to the lowest CBT value, the two parameters were highly correlated (see Figure 4; r = 0.77, n = 98, p < 0.01). While the difference in the two rhythms variability was large, Fisher's z-transformed values revealed that the amplitudes of the two parameters were similar. The amplitude of CBFV yielded a z score of 4.25 and CBT yielded a z score of 3.06.

### Blood pressure recordings and systemic hemodynamic variables

A Pearson correlation revealed a positive relationship between CBT and heart rate (r = 0.40, p < 0.01) across the 24 hour period. Diastolic blood pressure (DBP) and CBT showed a negative correlation (r = -0.30, p < 0.05). Et_CO2 _showed a trend towards a direct relationship with CBFV (r = 0.24, p = 0.10). Blood pressure, heart rate, and Et_CO2 _served only as regulators of CBFV and were not analyzed according to circadian phase.

## Discussion

This study is the first to use the constant routine (CR) protocol to determine whether the endogenous circadian pacemaker contributes to the previously reported diurnal changes in CBFV. The current work demonstrates that, with limited periodic external stimuli and a constant posture, there is 24-hour rhythmicity in CBFV. Subjects showed a cycle of approximately 24 hours in CBT, which has been previously demonstrated with the CR [21].

Figure 3 illustrates the intricate relationship between the rhythms across the study period. At approximately the CBT acrophase, the relationship between the two rhythms undergoes a transition. Between 180 and 240 degrees, CBFV is still rising and CBT is changing directions (first rising, reaching its peak and then falling). This period between 180 and 240 has been described as a "wake maintenance zone", a time in the circadian cycle during which humans are less likely to fall asleep [22]. In our subjects, the CBT is near its zenith or just starting to fall at this time and CBFV is still steadily rising. Higher values in CBT and CBFV are associated with activation and therefore these two endogenous rhythms may be promoting wakefulness during this "wake maintenance zone". However, at the end of this transition period, CBT is falling and CBFV is still rising, perhaps reflecting continued activation of the cerebral cortex. Whereas the two-process model predicts increased tendency to sleep as CBT falls [23], our finding may provide the mechanism by which wakefulness is effortlessly maintained before bedtime.

Figure 3 further illustrates that as wakefulness is extended past the subject's habitual bedtime (approximately 270 degrees), the two rhythms decline together. Between 0 and 60 degrees, CBFV steadily declines and CBT is steadily rising. The lower CBFV values in the morning may play a role in cognitive performance impairments [24], particularly the 3–4.5 hour phase difference in neurobehavioral functioning relative to the CBT rhythm that has been previously demonstrated in constant routine protocols [25].

Earlier studies using simultaneous EEG and TCD to continuously measure CBFV across the sleep period have concluded that, except for periods of REM sleep, [26,27], there is a linear decline in CBFV across the night during periods of non-REM sleep [1,28]. Other groups utilizing these techniques simultaneously speculated that the decline in CBFV through the night was a "decoupling" of cerebral electrical activity and cerebral perfusion during non-REM sleep [8-10]. In all studies [1,8-10,28], CBFV values were lower in the morning during wakefulness than during wakefulness prior to sleep at night. The current findings show that the decline in CBFV is present during wakefulness in the night time hours and therefore may not be attributed solely to sleep and associated changes that normally influence CBFV (including factors such as the shift to recumbency, and reduced activity, metabolic rate and respiratory rate).

Moreover, our interaction with the subjects and the monitoring of EEG for signs of sleep resulted in no sleep in all but one subject. The one exception was in a subject who lapsed into brief periods of sleep. Therefore, the fall in CBFV in 10 out of 11 subjects cannot be explained by the occurrence of non-REM sleep. It is possible, however, that the decline of CBFV across the night and early morning may be secondary to the sleep deprivation that is part of the constant routine. Brain imaging studies across sustained periods of wakefulness have shown significant decreases in absolute regional cerebral glucose metabolic rate in several areas of the brain [29-34].

The drop in CBT which preceded the parallel fall in CBFV needs to be considered as a possible explanation for the CBFV changes. The fall in CBT during sleeping hours is attributed in part to sleep-associated changes and in part to strong regular circadian forces independent of the sleep period. CBT is, in fact, one of the key and most extensively studied indices of the circadian phase. It is also known that CBT is highly correlated with brain temperature and brain metabolic rate [35]. Imaging studies have documented the intimate relation between brain activity and increased metabolic rate and oxygen delivery through perfusion. Therefore, it is plausible that CBT is a direct influence on CBFV or an index of decreased metabolic need for blood flow. The prevailing hypothesis that there is tight coupling of normal neuronal activity and blood flow was formulated over 100 years ago [36]. The drop in CBFV may be a consequence of the lowered cerebral activity secondary to lowered brain temperature. In contrast, two studies of exercise-induced hyperthermia showing decreased global and middle cerebral artery CBFV [37,38] do not support this hypothesized direct relationship between the two variables. However, one of the main purported mechanisms for the fall in CBFV in these exercise studies, the hyperventilation induced lowering of Pa_CO2_, is unlikely present during waking while lying in bed at night. Therefore, CBT declines remain a plausible explanation for the portion of the 24 hours when CBFV declined.

### Mechanisms of CBFV regulation

This protocol allowed the unique opportunity to evaluate blood pressure, heart rate, and Et_CO2 _in the absence of sleep, in subjects with constant posture, and highly restricted movements. While blood pressure clearly falls during sleep in normal individuals, the absence of sleep in the current study obviates the explanation that CBFV declines are secondary to lowered blood pressure. Furthermore, we sampled blood pressure throughout the day and night and found a weak inverse relationship between DBP and CBT. This finding is in contrast to a careful study of circadian influence on blood pressure in the absence of sleep which showed no change in blood pressure during the descending portion of the body temperature curve [39]. Nevertheless, our finding was weak and likely does not provide the explanation for the CBFV changes. The small-inverse relationship between Et _CO2 _and CBT is similar to that found by Spengler et al. [40], who showed a consistent but small amplitude circadian rhythm in mean end-tidal Et_CO2 _on a CR protocol. Et_CO2 _showed a trend towards a direct relationship with CBFV, which is consistent with previous studies showing that changes in Et_CO2 _are associated with changes in CBFV [41,42]. Heart rate was correlated with CBT, consistent with the findings of Van Dongen et al [39].

### Clinical correlation

The approximate 6 hour (90 degree) phase angle difference between the CBFV and CBT suggests that CBFV continues to decline into the early to mid-morning hours. This finding is consistent with a time window in the morning during which several physiological changes have been observed. For example, cerebral vasomotor reactivity to hypocapnia, hypercapnia, and normoventilation has been found to be most reduced in the morning [15,16]. It is tempting to suggest that the the low CBFV values in the morning may also help explain the well established diurnal variation of the onset of cerebrovascular accidents (CVAs) [43]. A meta-analyses of 11,816 publications between 1966 to 1997 found that there was a 49% increased risk of strokes between 6 am and 12 pm [44]. This time period is in agreement with studies on myocardial infarction (MI) and sudden death [45]. The increased incidence of these events has been attributed, in part, to the surge of blood pressure [13,46,47] and platelet aggregability [48,49] in the morning when patients are getting out of bed. Our results demonstrate that even in the absence of surges in blood pressure, the phase of CBFV reaches its lowest values during the hours before 12 pm. This further suggests that the endogenous rhythm of CBFV may be associated with the risk of CVAs in the late morning hours even without changes in posture or activity.

## Conclusion

Overall, the results demonstrate that CBFV, in the absence of sleep, exhibits properties of a circadian rhythm, as it rises and falls across a 24 hour period. The 6 hour (90 degree) phase angle difference in the CBFV rhythm with respect to the CBT rhythm may help explain previous findings of lower CBFV values in the morning. The phase difference occurs at a time period during which cognitive performance decrements have been observed and when both cardiovascular and cerebrovascular events occur more frequently. The mechanisms underlying this phase angle difference require further exploration.

## List of abbreviations

CBFV Cerebral Blood Flow Velocity

CBT Core Body Temperature

TCD Transcranial Doppler

EtCO2 End tidal Carbon Dioxide

DLMO Dim Light Melatonin Onset

EEG Electroencephalogram

MCA Middle Cerebral Artery

FFT Fast Fourier Transformation

CR Constant routine

EMG Electromyogram

SBP Systolic Blood Pressure

DBP Diastolic Blood Pressure

CVA Cerebrovascular accident

MI Myocardial infarction

## Competing interests

The author(s) declare that they have no competing interests.

## Authors' contributions

DAC coordinated, carried out, analyzed, and interpreted the study. AJS participated in the analysis and interpretation of the findings. DAC drafted the manuscript and AJS provided final approval of this version. RQS participated in data collection and data analysis. DAC and AJS co-designed the study. All authors read and approved the final manuscript.
